# The mediating role of psychological resilience and the moderating role of professionalism in the effect of mental wellbeing on anger and aggression in Olympic combat sports athletes

**DOI:** 10.3389/fpsyg.2026.1812239

**Published:** 2026-04-08

**Authors:** Zafer Gayretli, Emrah Secer, Mario Baic, Alisan Yavuz, Mustafa Can Koc, Ozkan Isik

**Affiliations:** 1 Department of Physical Education, Karadeniz Technical University, Trabzon, Türkiye; 2 Faculty of Sport Sciences, Erzincan Binali Yildirim University, Erzincan, Türkiye; 3 Faculty of Kinesiology, University of Zagreb, Zagreb, Croatia; 4 Faculty of Sport Sciences, Istanbul Yeni Yuzyil University, Istanbul, Türkiye; 5 Faculty of Sport Sciences, Istanbul Gelisim University, Istanbul, Türkiye; 6 Faculty of Sport Sciences, Balikesir University, Balikesir, Türkiye

**Keywords:** aggression, anger, combat sports athletes, mental wellbeing, professionalism, psychological resilience

## Abstract

**Purpose:**

This study aimed to examine the effect of mental wellbeing on anger and aggression among combat sports athletes and to investigate the mediating role of psychological resilience and the moderating role of professionalism in this relationship. The study sought to conceptually elucidate the associations between athletes’ psychological resources and their levels of anger and aggression.

**Method:**

The study was designed using a relational survey model and included 333 Olympic combat sports athletes (181 males, 152 females). Data were collected using the Warwick–Edinburgh Mental Wellbeing Scale–Short Form, the Competitive Aggressiveness and Anger Scale, the Connor–Davidson Resilience Scale–Short Form, and the Professionalism Scale for Athletes. The validity of the measurement models was tested through confirmatory factor analysis (AMOS 21). Hypotheses were tested using SPSS 25 and Hayes’ PROCESS Macro Models 4, 1, and 14.

**Results:**

The indirect effect of mental wellbeing on anger and aggression through psychological resilience was found to be significant (*b* = −0.302, 95% CI [−0.479, −0.148]), explaining approximately 9% of the total variance. The moderating effect of professionalism was examined, and the interaction between psychological resilience and professionalism was found to be statistically significant (*b* = −0.032, 95% CI [−0.051, −0.012]). Interaction analyses indicated that the effects were stronger at higher levels of professionalism. A conditional process analysis (PROCESS Model 14) revealed that professionalism moderated the indirect effect (*b* = −0.029, 95% CI [−0.047, −0.014]); specifically, the effect of mental wellbeing on anger and aggression through psychological resilience became more pronounced when professionalism was high.

**Conclusion:**

The findings demonstrate that mental wellbeing has a significant effect on anger and aggression among combat sports athletes through psychological resilience. This effect was stronger among athletes with higher levels of professionalism, highlighting its critical role in reducing anger and aggression. The results suggest that psychological resilience and professionalism are key factors in emotional regulation and performance management among athletes. Future studies may benefit from employing longitudinal and experimental designs to examine the effects of mental wellbeing on psychological resilience over time.

## 1 Introduction

Olympic Combat Sports (OCS) encompass disciplines such as boxing, fencing, judo, karate, taekwondo, and wrestling, which involve physical contact and require high levels of performance. Martial arts and combat sports are athletic activities governed by specific rules, in which techniques such as punching, kicking, or throwing are employed to gain physical superiority over opponents (Valdes-Badilla et al., 2022). By their nature, these sports expose athletes to physical and psychological stressors and require not only tactical and physical skills but also mental processes such as emotional regulation, stress management, and adaptation to challenging conditions (Predoiu et al., 2025; Ng-Knight et al., 2022; Santos et al., 2025). The high level of competition inherent in these sports increases the emotional burden on athletes, playing a decisive role in psychological expectations and performance both before and during competition. Although the number of studies in this field has increased in recent years due to the specific nature of combat sports, the literature still contains notable uncertainties (Barreira et al., 2025; Ciaccioni et al., 2025). Indeed, accidents, injury risks, and the physical presence of an opponent may increase the likelihood of emotional responses such as anger and aggression (Anderson and Bushman, 2002). Moreover, the literature emphasizes that athletes’ psychological adaptation processes influence not only performance outcomes but also ethical behavior (Havadar et al., 2024).

Anger and aggression may lead to different psychological outcomes depending on whether they are controlled, making them important variables for both athlete wellbeing and performance. Psychological wellbeing, a multidimensional construct encompassing life satisfaction, subjective wellbeing, psychological adjustment, and emotional balance, serves as a protective factor in regulating athletes’ negative emotional responses (Okuyucu et al., 2025). Mental strength, related to life satisfaction and emotional control, has also been shown to influence performance and psychological resilience in sport contexts (Uluç, 2023). Another prominent construct in the sport psychology literature is psychological resilience. Psychological resilience refers to an individual’s capacity to maintain psychological functioning and adapt successfully despite challenging circumstances (Yin et al., 2023). This concept is particularly important for understanding athletes’ psychological adaptation processes in high-stress sport disciplines such as OCS. Indeed, psychological resilience has been identified as a key determinant of emotional processes such as coping with stress among athletes (Kucuk Kilic, 2020). Furthermore, it has been emphasized that understanding how short-term stress reduction and long-term psychological benefits become functional in combat sports is of critical importance (Oh et al., 2025).

The role of psychological resources such as psychological resilience and mental wellbeing in reducing anger and aggression has been increasingly investigated. One study reported that psychological resilience and mental strength are associated with life satisfaction and mental health, whereas aggressive behaviors are linked to psychological wellbeing and self-control (de Lorenco-Lima et al., 2025a,b). Similarly, Secer et al. (2025) demonstrated that psychological resilience enhances life satisfaction. These findings indicate that psychological resilience plays a key role in emotional processes and in maintaining overall wellbeing. Within this framework, concepts such as professionalism and mental wellbeing are regarded as critical for understanding athletes’ emotional regulation, discipline, ethical behavior, and coping with competitive anxiety. In the context of combat sports, mental wellbeing has been shown to exert positive effects on both performance outcomes and psychological balance (Mojtahedi et al., 2023).

This study aimed to examine the effect of mental wellbeing on anger and aggression among athletes competing in OCS. It also sought to determine the mediating role of psychological resilience and the moderating role of professionalism in this relationship. The limited number of studies addressing emotional and psychological processes in combat sports enhances the potential theoretical and empirical contribution of the present study to the literature. Given the high demands of Olympic Combat Sports, mental wellbeing may provide athletes with psychological resources that help them manage stress and regulate emotions, potentially influencing anger, aggression, and resilience.

Mental wellbeing is a multidimensional indicator of mental health formed by the integration of psychological processes such as life satisfaction, psychological adjustment and emotional balance and it is widely accepted in sport psychology that this construct enhances athletes’ abilities to cope with stress (Ying and Yang, 2025). In sports characterized by high levels of physical contact and competition, such as combat sports, athletes are exposed to stressors including perceived physical risk, performance anxiety and direct confrontation with opponents before and during competition. These conditions create a fertile ground for the more frequent and intense emergence of emotional responses such as anger and aggression (Mojtahedi et al., 2023; Zekioğlu et al., 2024). While anger can negatively affect performance and ethical behavior when it becomes uncontrolled, aggression is a construct with both behavioral and emotional dimensions and involves the intention to harm others. Therefore, both are considered important research topics in terms of performance and the maintenance of a safe sporting environment (Mazzanti et al., 2025).

The positive psychology perspective posits that mental wellbeing is a process that enhances psychological resources, enabling individuals to manage negative emotions such as anger and to cope with the challenging aspects of life (Csikszentmihalyi and Csikzentmihaly, 1990; Seligman, 2011). This theoretical perspective aligns with Gross’s emotion regulation model, which emphasizes cognitive–behavioral strategies that increase or decrease the extent to which individuals respond adaptively or maladaptively to negative emotions. These strategies are used more effectively by individuals with higher levels of mental wellbeing (Gross, 1998). Empirical research on combat sports has highlighted that participation in martial arts and combat sports enhances self-esteem and emotional intelligence among young adults and students, positively contributing to overall life satisfaction, with mental wellbeing playing a decisive role in this process (Ying and Yang, 2025). In addition, studies conducted with Olympic combat sports athletes have shown that psychological resilience levels may serve as an indicator of a “warrior psychology” and may be associated with athletic performance (Predoiu et al., 2025). Other research has also reported positive relationships between mental wellbeing and psychological resilience among athletes, indicating that this relationship enhances athletes’ sport commitment and psychological functioning (Kül Avan and Parlakyıldız, 2024).

Systematic reviews and meta-analyses focusing on aggressive behaviors in combat sports athletes have shown that sport participation supports the reduction of aggressive behaviors through processes such as self-control, emotion regulation, and psychological adjustment, although direct effects are linked to complex mechanisms (Wang et al., 2025). These findings suggest that mental wellbeing is not only associated with positive psychological experiences but also constitutes a psychological resource that enhances athletes’ capacity to cope with negative emotional responses such as anger and aggression that arise within the sporting context. Building on the role of mental wellbeing in managing negative emotions, psychological resilience may further support athletes capacity to cope with stress and regulate anger and aggression, potentially acting as a mediating factor in these relationships.

Psychological resilience, derived from the Latin word “resilire” meaning “to bounce back,” refers to an individual’s ability to adapt to stressful situations, regulate negative emotions, and adjust to external stressors (Pânișoară, 2024; Xu et al., 2021). According to Resilience Theory, individuals can develop adaptive capacity through problem-solving and cognitive flexibility (Masten, 2001; Predoiu and Predoiu, 2024). In sport psychology, resilience is strongly associated with athletic success, psychological wellbeing, and emotional balance (Galli and Vealey, 2008; Yılmaz et al., 2024). In high-stress disciplines such as combat sports, psychological resilience enhances athletes’ emotional regulation, stress management, and performance-oriented behaviors (Predoiu et al., 2025; Fletcher and Sarkar, 2012). Empirical studies indicate that highly resilient athletes exhibit better anger control, lower emotional reactivity, and more adaptive responses under stress (Ozan and Secer, 2022; Çakmak Yıldızhan and Demir, 2024; Mei et al., 2025). Collectively, these findings support the mediating role of psychological resilience between mental wellbeing and anger/aggression, enabling athletes to manage negative emotional outcomes more effectively. Building on the potential mediating role of psychological resilience, athletes’ levels of professionalism may further influence how effectively resilience translates into adaptive emotional and behavioral responses. In other words, the positive effects of resilience on anger and aggression may vary depending on athletes’ professional attitudes, discipline, and ethical values, suggesting a possible moderating role for professionalism in high-stress combat-sport contexts.

Professionalism encompasses ethical values, discipline, responsibility, and professional attitudes, guiding athletes’ behavior in line with sport norms (Lumpkin et al., 1999; Güngör et al., 2022). In combat sports, high stress and competitive pressure make professionalism critical for managing anger, aggression, and ethical behavior while maintaining performance. Theoretically, Self-Determination Theory suggests that athletes regulate their behavior through autonomy, competence, and relatedness, thereby enhancing stress control and emotional regulation (Deci and Ryan, 2000). Professionalism supports psychological resilience, enabling athletes to respond adaptively to challenges and regulate negative emotions such as anger and aggression (Luthar et al., 2000; Fletcher and Sarkar, 2012). In this sense, professionalism serves as a moderating mechanism, strengthening the positive effect of resilience on emotional and behavioral regulation, particularly in high-stress combat-sport environments.

Given that combat sports involve high levels of competition and physical and psychological pressure, the effective use of mental wellbeing, psychological resilience, and professional behavior is of critical importance for athletes (Fletcher and Sarkar, 2012; Predoiu et al., 2025). While previous studies have examined the effects of mental wellbeing on anger and aggression and the mediating role of psychological resilience in this relationship, the influence of moderating factors such as professionalism has been addressed in a limited number of studies. Accordingly, the present study aims to examine the effects of mental wellbeing on anger and aggression among combat sports athletes and to identify the mediating role of psychological resilience and the moderating role of professionalism in this interaction. The study seeks to explain both the effects of psychological processes on sport performance and behavioral adaptation and the regulatory role of professional behavior.

The hypotheses of the study were formulated as follows:

H_1_: Mental wellbeing is negatively associated with anger and aggression indirectly through psychological resilience.

H_2_: Professionalism has a moderating role in the effect of psychological resilience on anger and aggression.

H_3_: The indirect effect of mental wellbeing on anger and aggression through psychological resilience depends on the moderating role of professionalism; that is, the effect of mental wellbeing on anger and aggression is more pronounced among athletes with higher levels of professionalism.

Taken together, the proposed hypotheses aim to clarify not only whether mental wellbeing is associated with anger and aggression in combat sports athletes, but also how and under what conditions this relationship operates. To address these questions, the present study adopted a relational survey design and tested a conditional process model in which psychological resilience was specified as a mediator and professionalism as a moderator. Accordingly, the methodological procedures described below were designed to empirically examine these hypothesized direct, indirect, and conditional effects.

## 2 Method

### 2.1 Research design

**Figure 1:**
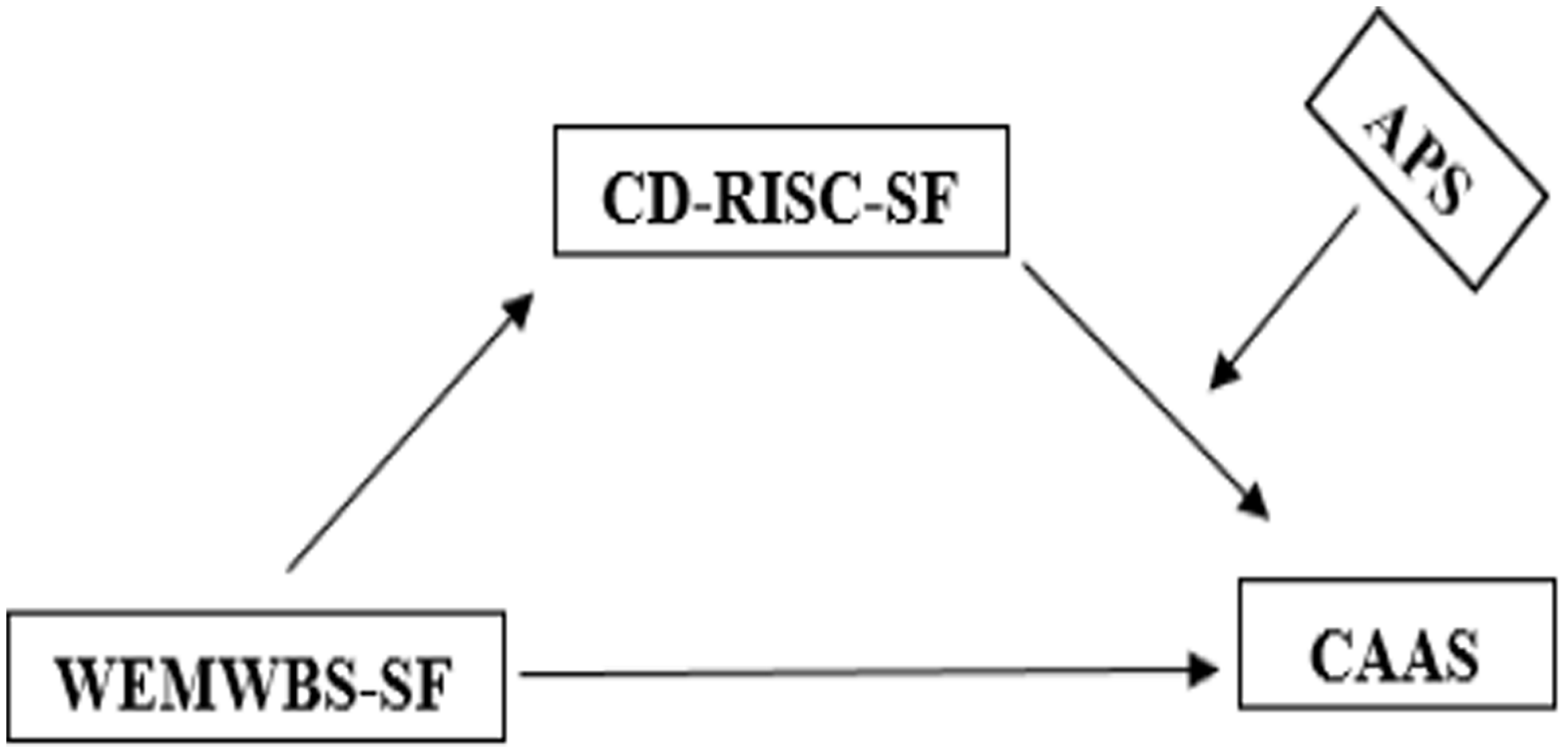
Research model.

This study was designed within the framework of the relational survey model, which is one of the quantitative research approaches aimed at examining the relationships between two or more variables (Christensen et al., 2015). In recent years, an increasing body of literature in the social sciences has emphasized that analyzing only direct relationships or unidirectional effects between variables is insufficient to explain the inherently multidimensional and complex nature of social phenomena. In this context, the importance of simultaneously considering moderating effects, which reveal the conditions under which relationships between variables are strengthened or weakened, and mediating effects, which explain the processes and mechanisms through which these relationships are formed, has been increasingly highlighted. Such integrated analytical approaches are considered to enable more comprehensive and in-depth testing of theoretical models and to provide original contributions to the literature (Gürbüz, 2021). The theoretical model tested within the scope of the present study is presented in Figure 1.

### 2.2 Participants

Criterion sampling, one of the purposive sampling approaches, was employed in determining the study group. This method is based on selecting individuals, situations, or cases that are directly related to the research problem and that meet predefined criteria (Büyüköztürk et al., 2024). The literature emphasizes that sampling criteria should be defined in alignment with the study’s overall aim and theoretical framework. This alignment is considered essential for ensuring methodological validity in the sampling process (Creswell and Clark, 2017). Accordingly, the inclusion criteria for participation in the study were defined as follows: (a) competing in one of the OCS disciplines, including boxing, fencing, judo, karate, taekwondo, and wrestling; (b) actively participating in the respective sport discipline; (c) providing voluntary consent to participate in the study; and (d) being 18 years of age or older. The exclusion criteria included participation in sport disciplines outside the scope of OCS, being under 18 years of age, having scale forms with incomplete or erroneous data, and cases with outlier values that violated the normality assumptions before statistical analyses. Based on these criteria, an initial sample of 383 athletes was included in the study. Following the exclusion of participants who met the exclusion criteria, the final analyses were conducted on data obtained from 333 athletes. The 333 athletes were recruited through national and regional Olympic combat sports federations and affiliated clubs. Coaches and sports directors shared the online Google Form with eligible athletes. All participants provided online informed consent and were explicitly informed of their right to withdraw from the study at any time without penalty. Data collection was conducted via the specifically prepared Google Form. The adequacy of the sample size was evaluated on the basis of both theoretical and statistical considerations. In this context, the sample of 333 athletes was considered sufficiently robust in terms of both statistical power and representativeness. Furthermore, according to the results of the power analysis conducted using G*Power software (*F* tests; linear multiple regression: fixed model, *R*^2^ increase; *f*^2^ = 0.08; *α* = 0.05; statistical power = 0.95; number of predictors = 3), a minimum of 219 participants was deemed sufficient. In this context, the sample consisting of 333 athletes was considered to be robust and reliable in terms of both statistical power and representativeness. Although participants varied in terms of training background and competition experience, no formal classification (e.g., amateur, semi-professional, elite) was systematically collected or included as a study variable. The demographic characteristics of the combat sports athletes participating in the study are presented in Table 1.

**Table 1:** Demographic characteristics of the combat sports athletes.

Variables		Female (*n* = 152)		Male (*n* = 181)		Total (*n* = 333)	
		Frequenc*y* (*n*)	Percentage (%)	Frequenc*y* (*n*)	Percentage (%)	Frequenc*y* (*n*)	Percentage (%)
OCS	Boxing	28	18.4	58	32.0	86	25.8
	Wrestling	19	12.5	33	18.2	52	15.6
	Judo	26	17.1	15	8.3	41	12.3
	Taekwondo	31	20.4	30	16.6	61	18.3
	Karate	40	26.3	22	12.2	62	18.6
	Fencing	8	5.3	23	12.7	31	9.3

	Min–Max	Mean ± SD	Min–Max	Mean ± SD	Min–Max	Mean ± SD
Age (year)	18–50	21.82 ± 6.03	18–58	23.55 ± 7.27	18–58	22.76 ± 6.78
Sports age (year)	1–39	10.86 ± 6.05	1–35	12.02 ± 6.80	1–39	11.49 ± 6.49
Body weight (kg)	40–104	60.01 ± 11.58	45–130	75.49 ± 14.43	40–130	68.43 ± 15.29
Body height (cm)	150–183	165.95 ± 6.63	158–193	177.86 ± 6.95	150–193	172.42 ± 9.05

### 2.3 Data collection tools

Data were collected after obtaining online informed consent from the participants, and they were explicitly informed of their right to withdraw from the study at any time without penalty. The data collection process was carried out via an online Google Form prepared specifically for the athletes.

#### 2.3.1 Warwick–Edinburgh mental wellbeing scale–short form (WEMWBS-SF)

The WEMWBS-SF, originally developed by Tennant et al. (2007), was adapted to Turkish culture by Demirtaş and Baytemir (2019). Within the Turkish adaptation study, the scale was found to consist of 7 items, employ a 5-point Likert-type response format and contain no reverse-coded items. Reliability analyses conducted during the adaptation process reported Cronbach’s alpha coefficients of 0.84 for the first study group and 0.86 for the second study group. These findings indicate that the scale is a valid and reliable measurement instrument for assessing levels of mental wellbeing within the Turkish cultural context.

#### 2.3.2 Competitive aggressiveness and anger scale (CAAS)

The CAAS, developed by Maxwell et al. (2009) to assess levels of anger and aggression in athletes, was adapted into Turkish by Gürbüz et al. (2019). The CAAS consists of 12 items rated on a 5-point Likert-type scale (1 = Strongly disagree, 5 = Strongly agree) and comprises two subscales—anger and aggressiveness—each containing 6 items. Higher scores obtained from the scale indicate higher levels of anger and aggression among athletes. Confirmatory factor analysis (CFA) conducted to test the construct validity of the scale demonstrated that the two-factor structure showed an acceptable fit to the data (*χ*^2^/df = 2.69, GFI = 0.91, AGFI = 0.87, CFI = 0.91, IFI = 0.91, SRMR = 0.07, RMSEA = 0.08). The Cronbach’s alpha coefficient of the scale was calculated as 0.83, while the Cronbach’s alpha coefficients for the anger and aggressiveness subscales were 0.70 and 0.84, respectively. These results indicate that the CAAS is a valid and reliable measurement tool for use in athlete samples.

#### 2.3.3 Connor–Davidson resilience scale–short form (CD-RISC-SF)

The CD-RISC-SF, developed by Connor and Davidson (2003), was used to assess individuals’ psychological resilience and coping capacities in the face of stress. The original form of the scale consists of 25 items and was subsequently tested in a university student sample by Campbell-Sills and Stein (2007), who adapted it into a 10-item short form. The Turkish adaptation of the scale was conducted by Kaya and Odaci (2021), and validity and reliability analyses revealed that the short form demonstrated a high correlation with the original 25-item long form. CFA results obtained during the Turkish adaptation indicated that the single-factor structure consisting of 10 items was validated in a university student sample. Reliability analyses reported a Cronbach’s alpha coefficients of 0.81 and a split-half reliability coefficient of 0.78. The CD-RISC–SF comprises 10 items under a single dimension and employs a 5-point Likert-type response format, with items rated from 0 = Not true at all to 4 = True nearly all the time. Total scores obtained from the scale range from 0 to 40, with higher scores indicating higher levels of psychological resilience and more effective coping strategies in dealing with stress, whereas lower scores indicate relatively weaker psychological resilience and greater difficulty in coping with stressful situations.

#### 2.3.4 Professionalism scale for athletes (APS)

The APS, developed by Güngör et al. (2022), is a unidimensional measurement instrument consisting of 11 items designed to assess athletes’ levels of professionalism. The scale uses a 5-point Likert-type response format, with responses ranging from “Strongly disagree” to “Strongly agree,” and contains no reverse-coded items. Total scores obtained from the scale range from 11 to 55, with higher scores indicating higher levels of professionalism among athletes. In the original scale development study, the Cronbach’s alpha coefficient was reported as 0.91 (Güngör et al., 2022). CFA conducted to examine the construct validity of the scale demonstrated that the single-factor structure exhibited an acceptable fit to the data (*χ*^2^/df = 2.705, RMSEA = 0.075, GFI = 0.931, AGFI = 0.896, and CFI = 0.931).

### 2.4 Validity and reliability of measurement models

To evaluate the construct validity of the scales used in the study, first-order and second-order CFA were conducted. Second-order CFA was applied to scales consisting of multiple subdimensions that were assumed to represent a higher-order latent construct. This approach allows for testing whether the subdimensions can be subsumed under a common higher-order structure. In contrast, for scales with a unidimensional structure comprising a single latent variable, first-order CFA was considered sufficient. This analytical strategy is consistent with the theoretical foundations of the scales and with measurement model practices recommended in the literature.

As the data met the assumption of multivariate normality, the Maximum Likelihood estimation method was employed in the CFA procedures. To achieve acceptable GFI indices as suggested in the literature, limited model modifications were applied where necessary, provided that they were theoretically justified. The model fit, validity and reliability levels of the scales used in the study were evaluated in detail, and the interpretation of GFI indices was based on the threshold values proposed by Byrne (2016) and Gürbüz (2021).

Convergent validity was evaluated using AVE and CR values. The AVE of the Aggressiveness and Anger Scale (CAAS) was 0.387, while CR values for all constructs exceeded 0.70, indicating good internal consistency. According to Hair et al. (2013), AVE values slightly below 0.50, such as 0.387 in the present study, can be considered acceptable when CR values are 0.70 or higher. This approach is also supported by Fornell and Larcker (1981), Hair et al. (2017), and Maruf et al. (2021), who note that AVE values above 0.40 may be sufficient if CR values are adequate. Model fit indices (*X*^2^/df, GFI, CFI, RMSEA, SRMR) were within acceptable ranges, further supporting the construct validity of the measures.

The internal consistency reliability of the scales was examined using Cronbach’s alpha coefficients. George and Mallery (2019) classified alpha coefficients above 0.80 as “good” and those above 0.90 as “excellent” reliability. Furthermore, Kalaycı (2018) indicated that alpha values ranging between 0.60 and 0.80 reflect acceptable reliability. In this regard, the reliability coefficients obtained in the present study were found to fall within the ranges recommended in the literature. All findings related to CFA, GFI, Cronbach’s alpha coefficients, Skewness and Kurtosis values, AVE and CR values for the scales used in the study are presented in detail in Table 2.

**Table 2:** Goodness-of-fit indices and threshold values of the scales, Cronbach’s alpha, normality analyses, AVE and CR values.

Index	Good fit	Acceptable	WEMWBS-SF	CAAS	CD-RISC-SF	APS
*X*^2^/df	<3	<3(*X*^2^/df) < 5	2.520	2.747	2.842	2.599
GFI	>0.95	>0.90	0.972	0.935	0.940	0.941
CFI	>0.95	>0.90	0.972	0.939	0.946	0.962
RMSEA	<0.05	<0.08	0.068	0.073	0.074	0.069
SRMR	<0.05	<0.08	0.034	0.059	0.050	0.037
Cronbach’s alpha	0.90	0.60–0.80	0.816	0.844	0.851	0.910
Skewness	–	−1/+1	−0.246	0.399	−0.541	−0.933
Kurtosis	–	−1/+1	−0.518	0.101	0.309	−0.028
AVE	>0.50	>0.40	0.402	0.387	0.387	0.491
CR	>0.70	>0.70	0.819	0.896	0.859	0.913

GFI = goodness-of-fit index; CFI = comparative fit index; RMSEA = root mean square error of approximation; SRMR = standardized root mean square residual; AVE = average variance extracted; CR = composite reliability.

### 2.5 Ethical approval

The study was approved by the Balıkesir University Health Sciences Non-Interventional Research Ethics Committee under decision number 2025/360. All research procedures were conducted in full compliance with the principles of the 1964 Declaration of Helsinki and international ethical standards.

### 2.6 Statistical analysis

Before data analysis, missing values and outliers were examined using SPSS 25.0. During this process, 50 data forms were excluded because they did not meet the inclusion/exclusion criteria, contained incomplete or erroneous data, or included outlier values that violated normality assumptions. Were excluded from the analyses. Accordingly, the dataset initially composed of 383 athletes was reduced to 333 athletes for the final analyses. At this stage, the assumption of multivariate normality was assessed, and the relationships among the independent, dependent, mediating and moderating variables were examined using Pearson correlation analysis.

The assumption of normal distribution was tested based on Mahalanobis distances, *Z* scores, and Skewness and Kurtosis values of the scale scores. Skewness and Kurtosis coefficients falling within the ±1 range (Hair et al., 2013) and *Z* scores within the −3 to +3 limits (Kalaycı, 2018) indicated that the data met the normality assumption. Linear relationships among variables were examined using scatterplots, and no significant deviations were observed. To assess the presence of multicollinearity, tolerance and variance inflation factor (VIF) values were examined; tolerance values greater than 0.20 and VIF values less than 10 indicated that multicollinearity was not a concern among the independent variables. CFA was conducted using AMOS 21 software to validate the factor structures of the measurement instruments used in the study. Following the confirmation of the factor structures, bootstrap-based regression analyses were performed to test the causal relationships among variables (Hayes, 2022). Hypotheses were tested using Hayes (2022) PROCESS Macro. Model 4 was used to examine the mediating effect, Model 1 to assess the moderating effect, and Model 14 to evaluate the conditional indirect effect. In all regression analyses, reliability was ensured through 5,000 bootstrap resamples (Hayes et al., 2017). For the model results to be considered statistically significant, the values obtained within the 95% confidence interval should not include zero (Hayes et al., 2017; Preacher and Hayes, 2008).

#### 2.6.1 Common method bias test

To assess the potential impact of common method bias, two diagnostic approaches were employed. First, Harman’s single-factor test was conducted on all measurement items using the Principal Axis Factoring method. This procedure was carried out in line with the recommendations of Podsakoff et al. (2024) to examine the presence of common method variance in the dataset. The results indicated that the single unrotated factor accounted for only 28.51% of the total variance. When compared to the widely accepted 50% threshold in the literature (Eichhorn, 2014), this value falls well below the cutoff, indicating that no single factor dominated the dataset and that the threat of common method bias was minimal. As an additional diagnostic approach, beyond the single-factor test, the correlation matrix of the latent constructs was examined. Although the correlations among variables were statistically significant, the highest correlation observed was *r* = 0.654. This value is substantially below the high threshold (*r* > 0.90) commonly cited in the literature as indicative of serious common method variance concerns (Podsakoff et al., 2024). Accordingly, both Harman’s single-factor test and the correlation-matrix-based diagnostic assessment support the conclusion that common method bias is limited in the present dataset.

## 3 Results

### 3.1 Correlation analysis and descriptive statistics

An examination of Table 3 indicates a negative and statistically significant relationship between WEMWBS-SF and CAAS (*r* = −0.245, *p* < 0.001), as well as positive and moderate statistically significant relationships between WEMWBS-SF and CD-RISC-SF (*r* = 0.654, *p* < 0.001) and between WEMWBS-SF and APS (*r* = 0.479, *p* < 0.001). In addition, negative and moderate statistically significant relationships were observed between CAAS and CD-RISC-SF (*r* = −0.298, *p* < 0.001) and between CAAS and APS (*r* = −0.395, *p* < 0.001). Finally, a positive and moderate statistically significant relationship was found between CD-RISC-SF and APS (*r* = 0.593, *p* < 0.001).

**Table 3:** Correlations among variables and descriptive statistics.

Variables	WEMWBS-SF	CAAS	CD-RISC-SF	APS	Mean	SD
WEMWBS-SF	1				28.15	4.35
CAAS	−0.245**	1			27.39	8.34
CD-RISC-SF	0.654**	−0.298**	1		29.30	6.23
APS	0.479**	−0.395**	0.593**	1	48.21	6.41

**p < 0.01.

### 3.2 Mediation and moderation analyses

To test the first hypothesis, mediation analysis based on PROCESS Model 4 (WEMWBS-SF → CD-RISC-SF → CAAS) was conducted. The bootstrap analysis results revealed that the indirect effect of mental wellbeing on anger and aggression through psychological resilience was statistically significant (*b* = −0.3025, 95% CI [−0.4794, −0.1484]). The variables included in the regression model explained approximately 9% of the total variance in anger and aggression. These findings indicate that the first hypothesis (H_1_) was supported.

To test the second hypothesis, a regression analysis based on PROCESS Model 1 was conducted to examine the moderating effect. The joint effects of psychological resilience (X), professionalism as the moderator (W) and their interaction term (X × W) on the outcome variable anger and aggression (Y) were evaluated. The results indicated that the direct effect of psychological resilience on anger and aggression was statistically significant (*b* = 1.412, 95% CI [0.4731, 2.3526]), whereas the direct effect of professionalism on anger and aggression was not statistically significant (*b* = 0.420, 95% CI [−0.1169, 0.9573]). The interaction term representing the psychological resilience × professionalism interaction (Int_1) was found to be statistically significant (*b* = −0.0321, 95% CI [−0.0513, −0.0129]). This finding indicates that professionalism moderates the relationship between psychological resilience and anger and aggression, functioning as a moderating variable. The variables included in the model explained 18% of the variance in anger and aggression.

**Figure 2:**
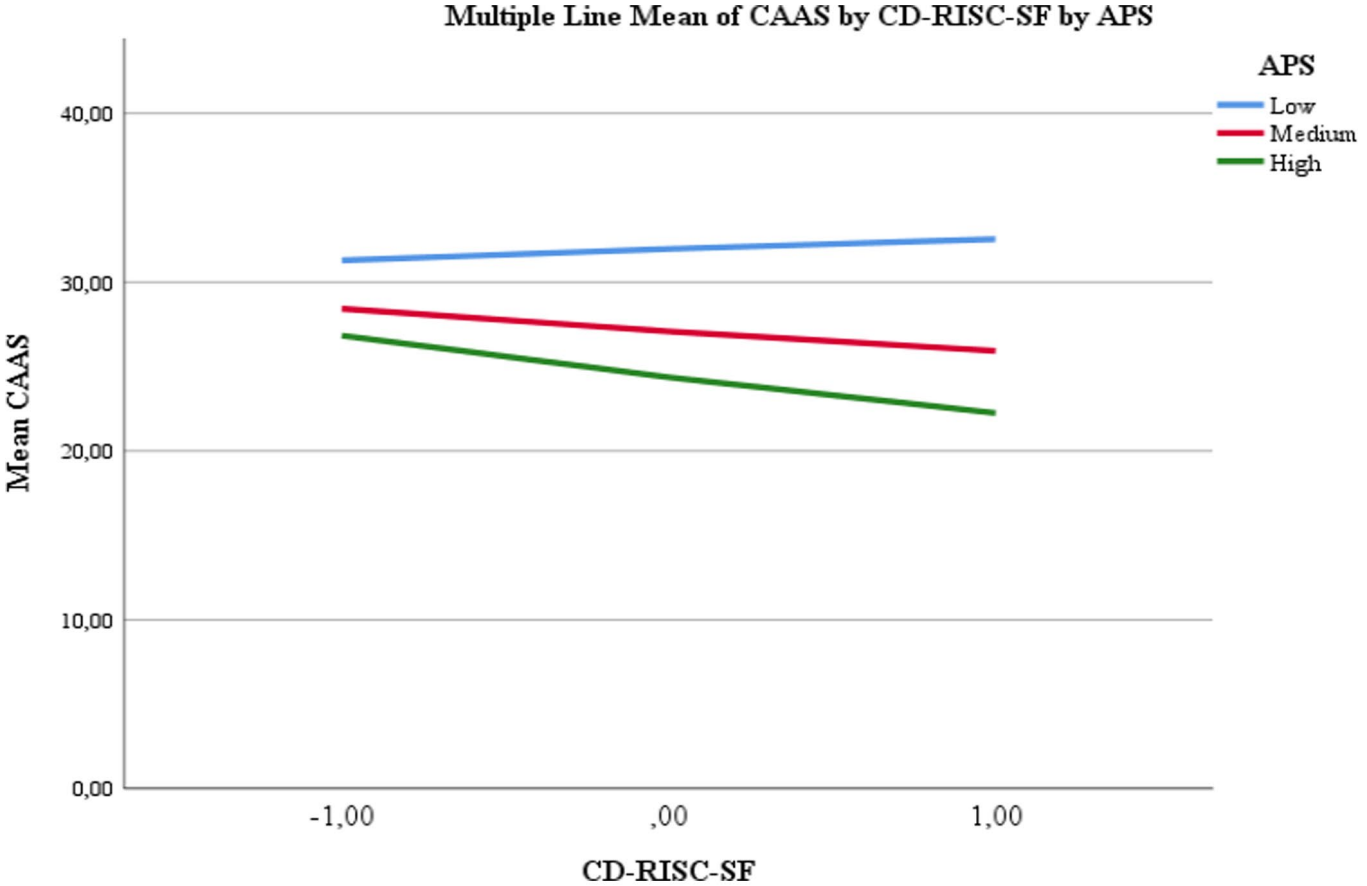
Graphical representation of the moderating effects.

To further examine the direction and magnitude of the moderating effect, a simple slopes analysis was conducted. The results showed that when professionalism was low, the moderating effect was not statistically significant (*b* = 0.0966, 95% CI [−0.1160, 0.3091]); however, when professionalism was at moderate (*b* = −0.1924, 95% CI [−0.3587, −0.0261]) and high levels (*b* = −0.3529, 95% CI [−0.5622, −0.1436]), the effect of psychological resilience on anger and aggression was statistically significant. These findings indicate that the second hypothesis (H_2_) was also supported. The figure illustrating this finding is presented below (Figure 2).

### 3.3 Conditional indirect effect (PROCESS model 14)

To test the third hypothesis, PROCESS Model 14 was specified, in which professionalism was examined as a moderator of the indirect (conditional) effect. The results of the conditional mediation analysis are presented in Table 4. The analysis conducted to determine whether the indirect effect of mental wellbeing on anger and aggression through psychological resilience was moderated by professionalism revealed that the index of moderated mediation was statistically significant (*b* = −0.029, 95% CI [−0.0472, −0.0145]). This finding indicates that professionalism plays a moderating role in this indirect effect and supports the third hypothesis (H_3_).

**Table 4:** Bootstrap regression analysis results.

Variables	Outcome: CD-RISC-SF (mediator)			Outcome: WEMWBS-SF			Outcome: CAAS (dependent variable)		
	*b*	LLCI	ULCI	*b*	LLCI	ULCI	*b*	LLCI	ULCI
Model 4 (H_1_)									
WEMWBS-SF (X)	0.936**	0.819	1.053	–	–	–	−0.166	−0.427	0.095
CD-RISC-SF (M)	–	–	–	–	–	–	0.323**	−0.505	−0.140
*R* ^2^	0.428						0.093		
Bootstrapt indirect effect	WEMWBS-SF ➔ CD-RISC-SF ➔ CAAS								
	*b* = −0.302, %95 CI [−0.479, −0.148]								
Model 1 (H_2_)									
CD-RISC-SF (X)	–	–	–	–	–	–	1.412**	0.473	2.352
APS (W)	–	–	–	–	–	–	0.420	−0.116	0.957
X*W (interaction)	–	–	–	–	–	–	−0.032**	−0.051	−0.012
*R* ^2^							0.188		
Predictors CAAS									
Model 14 (H_3_)									
WEMWBS-SF (X)	–	–	–	–	–	–	−0.027	−0.279	0.224
CD-RISC-SF (M)	–	–	–	–	–	–	1.414**	0.473	2.355
APS (W)	–	–	–	–	–	–	0.417	−0.120	0.956
X*W (interaction)	–	–	–	–	–	–	−0.031**	−0.051	−0.012
*R* ^2^							0.188		
Indirect effect	*b*	LLCI	ULCI						
Low APS	0.099	−0.093	0.310						
Medium APS	−0.169	−0.352	0.010						
High APS	−0.319**	−0.541	−0.100						
Moderated mediation index	−0.029**	−0.047	−0.014						

**p < 0.01; LLCI = lower limit confidence interval; ULCI = upper limit confidence interval.

**Figure 3:**
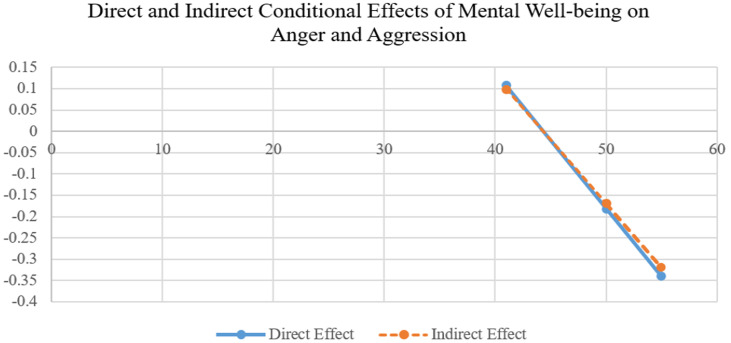
Graphical representation of the conditional mediation effect.

According to the results of the simple slopes analysis, the indirect effect of mental wellbeing on anger and aggression through psychological resilience was not statistically significant when professionalism was at low (*b* = 0.0995, 95% CI [−0.0933, 0.3106]) or moderate levels (*b* = −0.1695, 95% CI [−0.3527, 0.0101]). However, when professionalism was high (*b* = −0.3190, 95% CI [−0.5413, −0.1001]), the indirect effect of mental wellbeing on anger and aggression through psychological resilience was statistically significant. Overall, these findings indicate that the indirect effect of mental wellbeing on anger and aggression through psychological resilience among combat sports athletes is significant at high levels of professionalism, and that as professionalism increases, this effect becomes stronger in a negative direction, reflecting a greater reduction in anger and aggression (Figure 3).

## 4 Discussion

The primary aim of this study was to examine the effect of mental wellbeing on anger and aggression among OCS athletes and to test the mediating role of psychological resilience and the moderating role of professionalism in this relationship. The findings indicate that mental wellbeing indirectly affects anger and aggression through psychological resilience, rather than having a direct effect. Moreover, this indirect effect is significantly stronger among athletes with higher levels of professionalism. These results highlight the importance of conceptualizing psychological resources not as isolated constructs but as multidimensional mechanisms operating interactively, particularly in contexts such as combat sports characterized by high stress, intense competition, and heightened emotional arousal.

The findings supporting the first hypothesis demonstrate that mental wellbeing is negatively associated with anger and aggression through psychological resilience. This result suggests that mental wellbeing is not limited to the presence of positive emotions; rather, it constitutes a fundamental psychological resource that is related to individuals’ capacity to regulate emotional responses in the face of stress, threat, and frustration. Within the positive psychology framework, mental wellbeing is conceptualized as an integrated indicator of life satisfaction, psychological functioning, positive affect, and a sense of meaning, facilitating more balanced and adaptive responses to negative emotional stimuli (Diener et al., 2018; Huppert, 2014). In this context, individuals with higher levels of mental wellbeing are expected not only to experience more positive emotions but also to engage in more flexible cognitive appraisals in response to intense triggers of anger and aggression (Richard et al., 2023).

According to Gross’s (2015) process model of emotion regulation, individuals can regulate emotional responses either before or after they arise. Individuals with higher levels of psychological wellbeing tend to use adaptive strategies—particularly cognitive reappraisal—more effectively. This, in turn, reduces the likelihood that rapidly triggered emotions such as anger will translate into behavioral aggression. Nevertheless, divergent findings have also been reported. In athlete samples, psychological resources such as mindfulness are inversely associated with aggressive behaviors, with individuals high in mindfulness demonstrating lower aggression levels (Bolat et al., 2025). Recent studies further indicate that individuals with higher mental wellbeing display lower levels of aggressive behavior and more limited emotional reactivity under stressful conditions (Kok et al., 2013; Martínez-Monteagudo et al., 2019). Conversely, some research suggests that anger and aggression are related to impulsivity and attentional biases, implying that higher mental wellbeing may not translate into behavioral outcomes in the same manner for all individuals (Gröndal et al., 2023).

Similarly, within the sport psychology literature, mental wellbeing has been emphasized as an important predictor of aggressive behaviors and uncontrolled anger responses among athletes. It has been reported that aggressiveness and anger levels in sport are positively correlated with somatic and cognitive anxiety, which may have detrimental effects on performance (Kiratli, 2025). This evidence indicates that mental wellbeing may not always reduce aggression directly, underscoring the critical role of contextual factors and emotion management skills. Particularly in sport disciplines involving high competition and physical contact, low mental wellbeing may be related to perceptions of stress as a threat, which is associated with more aggressive behaviors (Ruiz et al., 2017). In contrast, athletes with higher mental wellbeing have been reported to interpret competitive pressure more as a challenge, enabling them to manage anger and aggression without converting these emotions into performance-incongruent behaviors.

In the context of combat sports, this relationship becomes even more critical. OCS such as boxing, judo, taekwondo, karate, wrestling and fencing inherently involve high levels of physical contact, intense emotional arousal and situations requiring rapid decision-making. In this context, athletes with lower mental wellbeing may be more prone to translating stress and frustration experienced during competition into anger and aggression, whereas athletes with higher mental wellbeing tend to maintain emotional balance and display sportsmanship- and control-oriented behaviors (Fawver et al., 2020; Uphill et al., 2014). Accordingly, the present findings indicate that mental wellbeing does not operate as a variable that directly suppresses anger and aggression in combat sports; rather, it functions as an indirect regulatory process operating through protective mechanisms such as psychological resilience.

The findings of the present study indicate that the effect of mental wellbeing on anger and aggression operates through psychological resilience rather than directly. This result suggests that psychological resilience constitutes a core mechanism that is associated with the translation of individuals’ positive psychological resources into functional outcomes under stressful and threatening conditions. Contemporary approaches conceptualize psychological resilience not as a static personality trait but as a context-sensitive and learnable process that develops through interactions between the individual and the environment (Gucciardi et al., 2015; Sarkar and Page, 2022). Theoretically, within stress–adaptation models, resilience is directly related to how individuals perceive, appraise, and regulate stressors. Modern stress models grounded in Lazarus and Folkman’s cognitive appraisal perspective suggest that highly resilient individuals tend to interpret stressful situations as “challenges” rather than “threats,” which is associated with lower intensity of negative emotional responses (Fletcher and Sarkar, 2016).

Moreover, resilience is not limited to direct positive effects; it also plays an important role in developing emotion regulation strategies and reducing aggressive response tendencies. Individuals with higher psychological resilience have been shown to be more effective in emotional reactivity and anger control (Ozan and Secer, 2022), indicating an association with reduced anger and aggression. While mental wellbeing strengthens the overall reservoir of psychological resources, psychological resilience functions as a transformative mechanism that enables these resources to be effectively mobilized under stress. Evidence regarding the mediating role of psychological resilience in sport psychology has been accumulating. Psychological resilience has been identified as critical in managing negative emotional responses related to stress and performance among athletes, indicating that resilience operates not only through direct effects but also via coping mechanisms and adaptation processes (Gupta and McCarthy, 2022).

At the same time, some findings suggest that the effects of psychological resilience are contextual and variable. One study reported that the impact of resilience on coping with stress, psychological pressure, and negative emotional arousal may vary significantly depending on sport discipline, competitive level, or individual differences (Wen et al., 2024). Studies have shown that resilience weakens the relationships between stress, anxiety, negative affect, and off-field maladaptive behaviors among athletes (Gucciardi et al., 2015; Bryan et al., 2019). Similarly, other research indicates that athletes with higher resilience regulate emotional arousal more effectively and display cognitively controlled behaviors rather than impulsive reactions (Levillain et al., 2024). These findings help explain why psychological resilience functions as a key mediator in the relationship between mental wellbeing and anger and aggression.

In combat sports, the mediating role of psychological resilience becomes even more salient. OCS continuously expose athletes to physical threat, direct contact with opponents, and intense competitive pressure. In such contexts, stress is inevitable, and the decisive factor is how athletes manage this stress. Recent empirical studies indicate that resilience in combat sports athletes is significantly associated with anger control, impulse regulation and sportsmanship-consistent behaviors (Xiang et al., 2025). Accordingly, it can be argued that athletes with higher mental wellbeing may, through psychological resilience, experience better regulation of anger and aggression under stressful competitive conditions. Demonstrating the mediating role of psychological resilience provides an explanatory mechanism for the relationship between mental wellbeing and anger/aggression variables, which are often examined separately in the literature. The findings suggest that mental wellbeing alone is related to aggression indirectly through protective processes such as psychological resilience. In this respect, the study suggests that interventions aimed at reducing anger and aggression among combat sports athletes should target not only mood states but also psychological resilience.

The findings related to the second hypothesis indicate that the effect of psychological resilience on anger and aggression differs significantly depending on athletes’ levels of professionalism. The results show that professionalism does not have a direct effect on anger and aggression; however, it functions as an important moderating variable that strengthens the effect of psychological resilience on these negative emotional outcomes. This suggests that professionalism is not merely a personality characteristic or a behavioral tendency but may provide a contextual framework that determines the conditions under which individual psychological resources become more functional.

The findings indicate that athletes at higher professional levels tend to utilize their psychological resilience more effectively, which is associated with better regulation of stress-related anger and aggression. This is consistent with the literature suggesting that psychological resilience functions as a “protective factor” in sport contexts; indeed, resilience has been found to be negatively associated with anger and aggression (Ozan and Secer, 2022). Theoretically, these findings align with Self-Determination Theory (SDT). Deci and Ryan (2000, 2012) argued that individuals’ capacity to regulate their behavior effectively depends on the satisfaction of basic psychological needs such as autonomy, competence, and relatedness. Athletes with higher professionalism are more likely to have internalized their athletic roles, structured their goals within a long-term career perspective, and integrated sport-related responsibilities with personal values. This internalization facilitates the effective use of personal resources such as psychological resilience in stressful and emotionally challenging situations (Gagné and Deci, 2005). Thus, it can be argued that in professional contexts, the relationship between psychological resilience and anger/aggression appears stronger. Evidence indicates that athletes with a professional and autonomous motivational profile develop more adaptive coping strategies in stressful competitive environments and regulate negative emotional responses more effectively (Amiot et al., 2004).

Conversely, when professionalism is low, resilience alone may not be sufficient to control anger and aggression, a possibility also discussed in the literature. The relationship between resilience and emotional responses may vary significantly depending on contextual factors (e.g., professional training, team discipline) (Li et al., 2025). Autonomous motivation has been reported to be positively associated with self-control, emotion regulation, and sportsmanship-consistent behaviors, whereas controlling forms of motivation show stronger associations with anger and aggression (Bartholomew et al., 2011; Ntoumanis et al., 2009). In this framework, professionalism may be considered a critical contextual factor that determines the extent to which athletes can translate resilience into behavioral outcomes. In addition, resilience research emphasizes that resilience gains functionality not in isolation, but through interaction with environmental and individual conditions (Luthar et al., 2000). Professional sport environments offer a more structured context through discipline, ethical norms, role expectations, and performance standards. This structured context may enable athletes with high resilience to regulate intense emotional responses such as anger and aggression more effectively (Fletcher and Sarkar, 2012). Indeed, this interaction between resilience and self-regulation is considered one of the fundamental determinants of emotional stability and behavioral control among elite athletes (Gucciardi et al., 2008; Sarkar, 2017).

When considered specifically in combat sports, this moderating effect becomes even more meaningful. OCS are among the disciplines with high risks of anger and aggression due to their structure involving intense physical contact, strong competitive pressure, and rapid decision-making. In this context, professionalism enables athletes to operationalize psychological resilience not only as “enduring adversity” but also as controlling impulsive reactions and keeping behavior within the boundaries of sportsmanship (Harwood et al., 2017). Accordingly, the findings provide theoretical and empirical explanations for why professionalism strengthens the relationship between psychological resilience and anger/aggression and under which conditions this relationship becomes more pronounced.

Findings related to the third hypothesis indicate that the effect of mental wellbeing on anger and aggression emerges through psychological resilience and that this indirect effect varies significantly depending on the level of professionalism. The results show that professionalism not only moderates the relationship between psychological resilience and anger/aggression but also functions as a critical contextual variable that conditions the extent to which mental wellbeing is translated into behavioral outcomes through psychological resilience. This suggests that professionalism shapes the functioning of psychological processes among combat sports athletes in a multilayered manner. In addition, the findings indicate that the effects of the mental wellbeing–psychological resilience relationship on aggression and anger do not remain constant in sport contexts, but vary depending on contextual conditions.

In particular, it has been reported that among combat sports athletes with high professional experience, the combination of mental wellbeing and psychological resilience regulates anger and aggression responses more effectively compared to athletes with lower training levels (de Lorenco-Lima et al., 2025a,b). This underscores the contextual role of professionalism in enabling athletes to use psychological processes more effectively. Theoretically, these results are highly consistent with Positive Psychology and Stress and Coping frameworks. Positive psychology conceptualizes mental wellbeing not merely as the presence of positive affect but as the effective use of psychological resources that enable individuals to cope with stressful and challenging life conditions (Ryff, 2013; Seligman, 2019). Individuals with higher mental wellbeing are known to make more flexible cognitive appraisals under stress, which strengthens psychological resilience (Fredrickson, 2001). However, the present findings suggest that this process does not operate in the same manner for all athletes and that professionalism determines the functionality of this chain.

Lazarus and Folkman’s (1984) Cognitive Appraisal and Coping Theory posits that how individuals appraise a stressful situation is a primary determinant of emotional and behavioral responses. Athletes with higher professionalism tend to appraise athletic challenges as more “controllable” and “development-oriented.” This cognitive framing not only facilitates the translation of mental wellbeing into psychological resilience but also strengthens the social and mental mechanisms through which psychological resilience mediates reductions in negative behavioral outcomes such as anger and aggression (Ciaccioni et al., 2024). Conversely, among athletes with lower professionalism, the translation of mental wellbeing into behavior via psychological resilience may remain more limited. Recent research in sport contexts emphasizes that the effects of psychological wellbeing on negative emotional outcomes related to performance depend on the structural characteristics of the context in which the athlete operates. In particular, among elite and semi-elite athletes, psychological wellbeing has been shown to reduce aggressive behaviors through resilience and self-regulation, and this relationship has been closely linked to the degree of internalization of the athlete role (Hill et al., 2018). In this respect, professionalism provides a framework that strengthens the protective effect of mental wellbeing by enabling athletes to internalize role expectations, ethical norms, and long-term goals.

When evaluated specifically in combat sports, this finding becomes even more critical. In disciplines characterized by high physical contact and competitive pressure, mental wellbeing alone may not be sufficient to reduce anger and aggression. However, the translation of mental wellbeing into behavioral regulation through psychological resilience becomes more feasible in contexts where professional norms are strong. This result indicates that aggressive behaviors among combat sports athletes should be considered not only in terms of individual psychological characteristics but also together with sport culture and the level of professional identity. Overall, the findings show that the mental wellbeing → psychological resilience → anger and aggression chain is not a linear or universal process; rather, it has a meaningful and protective function among athletes with higher professionalism. This supports the view—frequently emphasized in the sport psychology literature—that psychological resources are contextually activated and highlights the importance of strengthening professional identity in psychological interventions for combat sports athletes. To translate these findings into practice, mental health-supportive programs should be developed, including Cognitive Behavioral Therapy (CBT) sessions focused on anger and aggression management, as well as professionalism workshops designed to enhance role internalization, ethical behavior, and self-regulation under competitive pressure. These interventions can help athletes effectively utilize their psychological resources, particularly in high-stress combat sports contexts.

### 4.1 Strengths, limitations, and future directions

#### 4.1.1 Strengths

One of the main strengths of this study is that it examined the relationships between mental wellbeing, anger, and aggression in athletes competing in OCS not only at the level of direct effects but also within a comprehensive model incorporating psychological processes and contextual factors. Testing psychological resilience as a mediator and professionalism as a moderator within the same model provides a more nuanced understanding of the emotional and psychological dynamics of individuals in sport disciplines characterized by intense competition and substantial physical contact. Theoretically, the study demonstrates within the Positive Psychology framework that mental wellbeing strengthens individuals’ resilience and coping capacities, while Stress and Coping Theory provides a foundation for explaining the mediating role of psychological resilience in the relationship between mental wellbeing and anger/aggression. In addition, from a Social Cognitive Theory perspective, professionalism offers insight into how athletes regulate psychological resources and how their behavioral responses are shaped under high-stress conditions. Together, these frameworks clearly show that emotional and behavioral responses observed among OCS athletes arise not only from individual characteristics but also from the interaction of psychological processes and contextual factors.

The sample structure also represents an important advantage. Including individuals actively participating in OCS disciplines such as boxing, fencing, judo, karate, taekwondo, and wrestling enhances both the theoretical and practical value of the findings. In this context, the study more clearly elucidates the role of psychological processes in OCS disciplines, where anger and aggression are intertwined with performance and occur under high stress. Methodologically, comprehensively testing the construct validity and reliability of the measurement instruments strengthened the robustness of the measurement model. Confirming factor structures, rigorously examining normality assumptions, and evaluating common method bias indicate that the results are grounded on a methodologically reliable basis. Finally, the statistical analytical approach constitutes a major strength. Systematically testing mediation, moderation, and conditional mediation effects within the same dataset clarified not only the presence of relationships among variables but also the conditions under which these relationships are strengthened or weakened. This provides a meaningful theoretical and applied contribution to understanding the psychological dynamics of OCS athletes.

#### 4.1.2 Limitations

This study also has several limitations. First, because the study employed a cross-sectional design, it is limited in establishing causality among variables. Although the observed effects among mental wellbeing, psychological resilience, and anger–aggression were significant, longitudinal designs are needed to test changes and directionality over time, and qualitative and mixed-method designs are required for more in-depth examination. Second, the sample was limited to athletes actively participating in OCS disciplines, which may restrict the generalizability of the findings to other sports or the general population. In particular, the high levels of physical contact and competition inherent in combat sports may differentiate anger and aggression responses. Therefore, the results may reflect psychological processes that are specific to similar sport disciplines.

A third limitation is that data were collected online using self-report scales. Responses based on participants’ self-perceptions may have been influenced by social desirability or self-presentation tendencies. Although common method bias analyses indicated minimal risk, future studies could enhance validity by incorporating objective measures or observational data. Finally, while the study examined the effects of psychological resilience and professionalism, other individual and environmental variables were not included. Considering these variables may provide a more detailed explanation of the relationship between mental wellbeing and anger–aggression in OCS athletes and improve the explanatory power of the model.

#### 4.1.3 Future directions

This study has contributed to the literature by examining the relationships among mental wellbeing, psychological resilience, professionalism, and anger–aggression in athletes competing in OCS. Based on the findings, several directions for future research are suggested:

Given that cross-sectional findings limit causal inference, future research may employ longitudinal designs to track the effects of mental wellbeing on psychological resilience over time and to examine how these processes shape anger and aggression. In addition, experimental designs implementing different intervention programs may be conducted to test the effectiveness of strategies aimed at enhancing psychological resilience and reducing aggressive responses among OCS athletes.

Future studies may investigate the effects of individual and contextual factors such as social support, coaching style, training load, and level of competition. Incorporating these variables would enable more comprehensive modeling of the psychological processes of OCS athletes and strengthen practice-oriented recommendations.

As the present study focused exclusively on OCS athletes, conducting comparative studies with athletes from other sport disciplines may help clarify the unique psychological dynamics of combat sports and the specific role of professionalism in these contexts.

This study examined the relationships between mental wellbeing and psychological resilience within the frameworks of stress and anger management theories. Future research may adopt expanded models integrated with other psychological theories—such as anxiety, motivation, or self-regulation theories—to gain a deeper understanding of OCS athletes’ performance and psychological wellbeing.

The findings may serve as a guide for coaches and sport psychologists. Future studies could test the effectiveness of mental health–supportive intervention programs and professionalism-focused training in reducing aggression and enhancing psychological resilience among OCS athletes.

## 5 Conclusion

This study comprehensively examined the relationships among mental wellbeing, psychological resilience, professionalism, and anger–aggression levels in athletes competing in OCS. The findings revealed that mental wellbeing plays a critical role in reducing anger and aggression by strengthening athletes’ psychological resilience. Moreover, this indirect effect was more pronounced among athletes with higher levels of professionalism, confirming that professionalism functions as a moderating factor in the relationship between psychological resilience and anger–aggression.

Within the framework of positive psychology, the study contributes to understanding how mental wellbeing influences individuals’ psychological resilience, while stress and anger management theories provide a robust theoretical basis for explaining how psychological resilience and professionalism shape aggressive behaviors. This theoretical integration offers valuable insights for designing interventions that support both the individual psychological processes and sport performance of OCS athletes. The findings not only contribute theoretically to the literature but also offer practical implications for OCS athletes. In particular, educational programs aimed at enhancing mental wellbeing and psychological resilience, as well as development strategies focused on professionalism, may help athletes better manage aggressive tendencies and optimize performance. Furthermore, the results indicate that OCS disciplines reflect distinct psychological dynamics and may require models that differ from those applied to other sport disciplines.

In conclusion, by providing a holistic explanation of the multidimensional processes influencing the psychological wellbeing and behavioral responses of OCS athletes, this study offers a valuable reference framework for both scientific research and applied practice. Future research may further advance understanding of psychological resilience and professionalism development among OCS athletes by applying this model across different sport disciplines, age groups, and cultural contexts.

## Data Availability

The raw data supporting the conclusions of this article will be made available by the authors, without undue reservation.
